# London as an Educational Centre

**Published:** 1907-09-07

**Authors:** 


// The Hospital

A Journal of

The Medical Sciences and Hospital Administration.

New Series. No. 24, Vol. I. [No. 1096, Vol. XLII.] SATURDAY, SEPTEMBER 7, 1907.

### EDUCATIONAL NUMBER.

### LONDON AS AN EDUCATIONAL CENTRE.

In view of its commanding position in relation to so many forms of human thought and action, it would be strange indeed were London not able to claim a prominent place among the medical schools of the country. Though it must, perhaps, be admitted that everything is not yet exactly what educationists could wish, it still remains true that in some respects London is not only prominent, but is even pre-eminent.

The great teaching hospitals of the metropolis can boast a proud and distinguished history, not only in reference to their primary duty—namely, the treatment of the sick and injured—but also as centres of scientific, medical and surgical research. The staffs of these hospitals, both in the past and at the present day, include names which are "writ large" in the story of professional advance. In the wards and out-patient departments is to be found a collection of clinical material suitable for the purposes of the student such as cannot be rivalled in any part of the Empire. And among the clinical teachers of the metropolis are names familiar as household words wherever scientific and practical medicine is known and practised. It is inevitable, seeing what London is in the political and in the social world, that numbers of highly competent men shall be attracted within her borders. This is true of all forms of human activity. It is true of politics, of divinity, and of law. It is also true of medicine. Hence, without in the least instituting invidious comparisons, London may justly and appropriately claim the possession of larger numbers of distinguished physicians and surgeons and of experienced clinical teachers than any other city. And when, in addition, the abounding opportunities for practical study offered by the larger hospitals are considered, it is manifest that as a school of clinical medicine and clinical surgery the metropolis occupies a special and even a unique position. One other remark on this point may be permitted. The leading medical schools here are not the growth of yesterday. They have long been devoted to the training of medical students. Hence, apart from other advantages, they possess rich and valuable museums, and these are of the greatest importance in the scheme of medical education. To this statement there may also be added the further fact that many of the sciences ancillary to medicine possess in London central institutions where there are special opportunities for theoretical and practical work, and that in all departments there are facilities particularly suited to the industrious and ambitious student.

It must, however, be recognised that, at least in the past, London has not altogether succeeded in organising victory to an extent commensurate with her great opportunities. There has been too absolute a divorce between the plan of medical education and the scheme of medical examination, and the total number of students entering at the metropolitan schools has for some years been declining. This is not the place in which to inquire the exact reason for such a condition, but it is certain that the matter has attracted the attention of those in authority. Further, whether the plan of organisation now adopted by the University of London is or is not sound, there is no question that it will do something to improve the teaching of the earlier subjects of the medical curriculum in certain of the metropolitan schools. And even if some of the smaller schools are led to abandon competition in these departments, the remainder may count on more adequate equipment and on enlarged opportunity. Still, the present state of matters does call for particular care on the part of those who are about to enter on their medical studies in London. It is of the first importance that the student at the very outset should clearly define his aim—that is to say, he should have a definite view as to whether he hopes to take a University degree, or whether, on the other hand, he will be content with a diploma or license from the Colleges of Physicians and Surgeons. Nothing is more annoying to a capable student to find out, perhaps near the end of his curriculum, that he is prevented from presenting himself for the higher qualifications because he failed in earlier days either to pass a particular preliminary examination or to attend certain special classes in the preliminary sciences. It is impossible to urge the advice just given in too strenuous terms. Indeed, there can be little doubt that neglect of a clear perception on this point has damaged the cause of medical education in the metropolis. Too late have students discovered that the curriculum they have adopted has confined their ambitions to a mere license to practise, and has left closed against them, probably for all time, the distinction of a University degree. This consideration, further, is to-day even of more importance than it was in the past. There is an increasing recognition of the advisability of the doctor being a University graduate, and having the unquestioned right to the title "M.D." Therefore, again, we advise the student who cultivates this aim —and all should do so as far as possible—to see that his start is in accordance with his ultimate desire. If he is safe here, he may feel certain that the steady application, even of ordinary ability, and the unwearied use of opportunities, particularly of those which mean practical and direct personal experience, will lead him to success. And with London University as his *alma mater* he may certainly claim an academic distinction of the very first rank.

